# Short-term intra-arterial infusion chemotherapy for head and neck cancer patients maintaining quality of life

**DOI:** 10.1007/s00432-018-2784-4

**Published:** 2018-10-31

**Authors:** Karl R. Aigner, Emir Selak, Kornelia Aigner

**Affiliations:** Department of Surgical Oncology, Medias Klinikum Burghausen, Krankenhausstr. 3a, 84489 Burghausen, Germany

**Keywords:** Head and neck cancer, Intra-arterial chemotherapy, Quality of life, Regional chemotherapy

## Abstract

**Purpose:**

Head and neck cancer treatment achieves good locoregional tumor control rates while causing severe side effects. Therapy with chemotherapeutic drugs administered intravenously is limited because either the concentrations at the tumor site are too low or the total dosages are too high. The evaluation of a technique for short-term intra-arterial infusion chemotherapy is described herein.

**Methods:**

In a retrospective study, we reviewed the medical records of 97 patients with head and neck cancers who received short-term intra-arterial infusion chemotherapy (62 patients previously untreated, 35 patients with prior radiotherapy). All patients refused further radiotherapy. Response rates, overall survival and adverse effects were the study endpoints. The blood supply of the tumors was controlled with indigocarmine blue infusion and staining of the tumor region.

**Results:**

Complete or partial response was found in 67%, 52% and 63% of previously untreated patients and in 25%, 30% and 29%, respectively, of previously irradiated patients for staging groups I–III, IVA and IVB/C. Patients with T3/T4 tumors who were previously irradiated showed a median overall survival of 9 months, and those without pretreatment showed a median overall survival of 22.5 months. None of the patients required tube feeding. No new case of dysphagia, xerostomia, or functional speech and hearing loss was reported. Pain and clinical symptoms were reduced for all patient groups. Indigocarmine staining showed reduced tumor blood supply in previously irradiated regions but good blood supply in untreated regions.

**Conclusions:**

Short-term intra-arterial infusion chemotherapy achieves promising response rates and lacks severe adverse effects.

**Electronic supplementary material:**

The online version of this article (10.1007/s00432-018-2784-4) contains supplementary material, which is available to authorized users.

## Introduction

### Current treatment options, adverse effects and suicide rates

The standard head and neck cancer therapies, high-dose radiation accompanied by intravenous cisplatin chemotherapy, lead to satisfying tumor control rates but are often limited because of aggravating adverse effects. Dysphagia, tracheostomy, mucositis, weight loss, functional speech and hearing loss often result from radiotherapy(Achim et al. 2017). The suicide risk for head and neck (HN) cancer patients is greater than for other cancer incidences and is increased in patients treated with radiation alone compared to those treated with surgery alone (Green and Griffiths 2014; Anguiano et al. 2012; Misono et al. 2008; Osazuwa-Peters et al. 2016). The reasons for the increased suicide rates in HN cancer patients are poor quality of life due to the therapy’s side effects (Park et al. 2016; Briscoe and Webb 2016; Ringash et al. 2015; Zecha et al. 2016). Radiation’s side effects derive from damage to healthy tissue including the facial nerves, and systemic chemotherapy’s side effects mainly derive from cisplatin toxicity.

The control of tumors that positively test for human papilloma virus (HPV) seem to be achieved more easily and is, therefore, under investigation for a more gentle treatment, such as intensity-modulated radiotherapy (IMRT) or transoral robot surgery (TORS), without primary chemoradiation (Marta et al. 2014). Only R0 resections give hope for a possible omission of radiochemotherapy.

However, the problem of severe side effects not only remains but increases while tumor control improves in general. A shift in the patient cohort to more HPV-related tumors with a better prognosis may lead to a more gentle treatment with less radiotherapy. For the younger generation, there is an even more urgent need for treatment without life-threatening or sociopsychologically problematic adverse effects (Ringash 2015; Rathod et al. 2015). Irradiation for HNC accounts for good locoregional tumor control rates but is also the main reason for severe side effects and patients’ discomfort. Chemotherapy administered intravenously is limited mainly because of its nephrotoxicity. An efficacious concentration at the tumor site would require a non-tolerable systemic dosage. We, therefore, investigated the feasibility and response rates of intra-arterial infusion chemotherapy and observed drug concentrations in the tumor-supplying artery as well as the tumor-draining vein.

### Chemotherapy application methods

The standard application method for chemotherapeutic drugs is intravenous infusion. The infusion time ranges from 30 min to several hours. The drug is distributed in the whole-body blood volume, and the drug concentration at the tumor site is similar to that in the rest of the body.

An alternative application method is intra-arterial (i.a.) infusion, where the drug is administered via an angiocatheter or an implanted port catheter that allows infusion into the tumor-supplying artery and, in the case of head and neck cancer, the carotid artery. Different techniques of infusion result in different drug exposure times and concentrations reached at the tumor site. An i.a. bolus injection, usually applied with very high cisplatin dosages (100 mg/m^2^), reaches high local concentrations during a brief infusion time of less than 1 min, which are decreased by thiosulfate (Robbins et al. 2010; Kovacs et al. 2012). An i.a. short-term infusion differs from a bolus injection in terms of dosage (maximum 55 mg/m^2^) and infusion time (5–12 min). Drug exposure rates for these three applications differ from each other, as i.a. bolus injection yields very high drug concentrations (55,000 ng/ml) in less than one minute, and i.a. short-term infusion yields relatively high drug concentrations (25,000 ng/ml) in approximately 12 min. The advantages of intra-arterial chemotherapy for head and neck cancer treatment include increased drug concentration at the tumor site with decreased systemic drug levels in the rest of the body. Decisive results of i.a. chemotherapy concerning locoregional and distant tumor control and possible side effects have remained unavailable mainly for two reasons. First, the combination with radiation does not give a clear picture of which effect (tumor control or adverse) is derived from radiation and which is derived from chemotherapy. Quality of life studies comparing i.a. chemoradiotherapy versus intravenous (i.v.) chemoradiotherapy are affected by radiation as the main source of adverse effects. Second, the exact technique of i.a. infusion is crucial for the outcome, and former studies have not always been optimal in terms of catheter position, concentration, and time of chemo exposure (Robbins et al. 2010; Kovacs et al. 2012; Rasch et al. 2010).

## Methods

### Patient description

This is a retrospective observational cohort study. We reviewed the medical records of 97 patients with head and neck cancers who received short-term intra-arterial chemotherapy within 1992–2017. Observation time was minimum 10 months, with a median of 34 months.

Inclusion criteria were nasopharyngeal (*n* = 8), hypopharyngeal (*n* = 19), and oropharyngeal (*n* = 70) carcinoma. All patients who received short-term intra-arterial chemotherapy had either refused treatment with systemic chemotherapy and radiotherapy at any time (*n* = 62) or received prior treatment (*n* = 35). Investigations were performed in compliance with the principles of good clinical practice outlined in the Declaration of Helsinki and federal guidelines, and had approval by the Medias Institutional Review Committee. Informed consent was obtained from each participant or participant’s guardian.

Treatment techniques differed according to tumor extension and individual feasibility. Sixteen patients received intra-arterial (i.a.) chemotherapy through an angiocatheter, 13 of which with additional chemofiltration. Nineteen patients received i.a. chemotherapy through an implanted intra-arterial port catheter, 9 of which with additional chemofiltration. 5 patients received a sequence of therapies with altering techniques of angiocatheter and implanted port catheter, 4 of which with additional chemofiltration. Fifty seven patients received isolated thoracic perfusion in addition to the port- or angiocatheter technique. The total number of i.a. chemotherapy procedures was 500, out of which 126 were angiocatheter techniques, 214 were administered via port catheters and 160 had additional isolated thoracic perfusion. Median number of treatment cycles per patient was 4.5. Two patients received irradiation after i.a. chemotherapy. The patients were divided into subgroups according to prior treatment and staging. Patients without any pretreatment and patients with prior radiotherapy or radiochemotherapy were separated. Further partition was dependent on staging. Staging groups included I–III, IVA, and IVB/C. A detailed patient description is shown in Table 1.

**Table 1:** Head and neck cancer patient distribution according to their staging and if prior irradiation was applied

Stage	all	I	II	III	IVA	IVB	IVC
Total	97	2	9	5	59	5	17
Prior radiotherapy	35	1	1	2	17	3	11
No radiotherapy	62	1	8	3	42	2	6

Patients without prior irradiation did not receive any other treatment prior to intra-arterial short-term infusion chemotherapy

The median age was 59 years (range 35–84), and there were more male (*n* = 67) than female (*n* = 58) patients; however, the median age was similar. The largest subgroup was advanced stage HNC IVA with 59 patients (17 with prior radiotherapy, 42 without any prior therapy). Stages I–III were merged into one subgroup with 16 patients (4 with prior radiotherapy, 12 without any prior therapy). Stages IVB and IVC were merged into one subgroup with 22 patients (14 with prior radiotherapy and 8 without any prior therapy). The median observation time was 39 months (minimum 10 months).

### Short-term intra-arterial infusion techniques

Several techniques for short-term intra-arterial drug delivery were developed. They all allow for the direct infusion of chemotherapeutic drugs into the tumor-supplying artery and all are combinable with drug filtration, which in most cases is applied. A major criterion of short-term intra-arterial infusion is the infusion time of 5–12 min.

#### Intra-arterial port system (i.a. port) for short-term infusion

A JetPort-Allround catheter (PfM, Cologne, FRG) is implanted into the common carotid artery, which allows for repeated infusions without further surgery. Drugs can easily be infused intra-arterially through the port. The infusion time is 5–12 min. Drug filtration can be conducted at the same time in the subclavian vein with a Sheldon catheter.

#### Short-term intra-arterial infusion through an angiocatheter

For arterial infusion through an angiocatheter, the catheter is inserted into the femoral artery in the groin under local anesthesia, and its tip is directed into the common carotid artery under X-ray monitoring. For drug filtration, a double channel central venous access is used (Sheldon catheter).

#### Short-term intra-arterial (i.a.) infusion via the port catheter or angiocatheter combined with isolated thoracic perfusion (ITP)

**Fig. 1:**
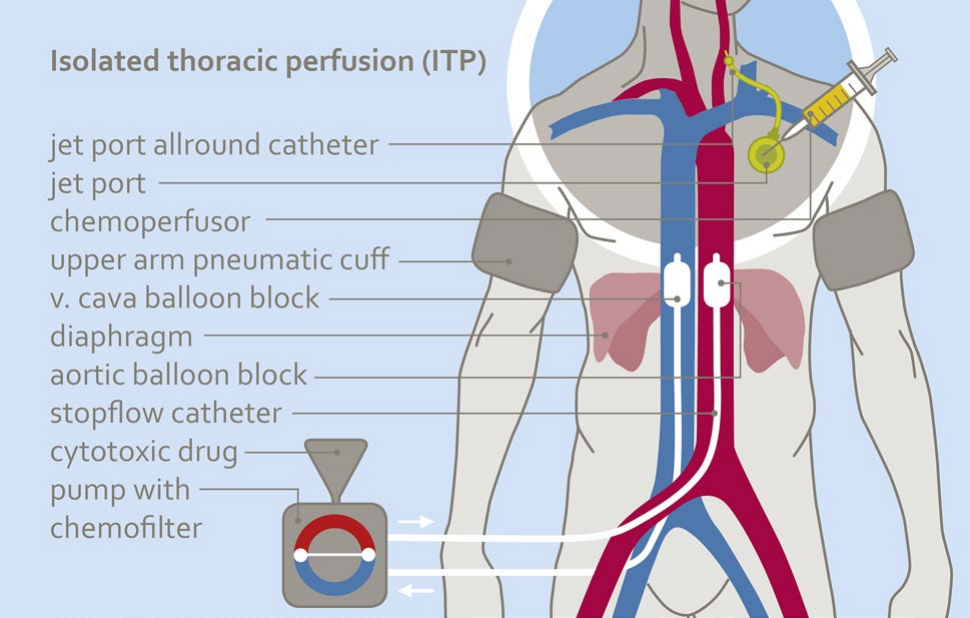
Scheme of isolated thoracic perfusion combined with intra-arterial short-term infusion via a jetport allround catheter. Due to the reduced circulating blood volume, tumors are exposed to higher concentrations of cytotoxics

In addition to an i.a. port infusion or angiocatheter infusion of chemotherapeutics (as described in 1. and 2.), an isolated thoracic perfusion (ITP) can be applied to further increase drug concentrations at the tumor area without increasing the dosage. Drug infusion is administered through the angiocatheter inserted via the femoral artery and ITP is conducted with a stopflow balloon catheter (Dispomedica, Hamburg, FRG) that stops the blood flow with balloons in the aorta and vena cava. Blood flow is stopped contemporarily with infusion time and is continued several minutes after the infusion ends. The total time of ITP is 15 min (Fig. 1). For drug filtration, the perfusion channels of the stopflow balloon catheters were used.

#### Technique selection and drug regimen

Patients without or only minor pretreatment and up to WHO stage IVB received i.a. chemotherapy via angiocatheters or implanted port catheters according to the individual feasibility. Heavily pretreated patients and patients with distant metastases were submitted to additional ITP.

Drug combinations for short-term i.a. chemotherapy with chemofiltration were cisplatin (40–50 mg), adriamycin (15–30 mg) and mitomycin C (10–15 mg) per treatment cycle (5 to 12 min infusion time, treatment cycles in three weeks intervals). If i.a. chemotherapy is applied without chemofiltration, lower dosages of cytotoxics are used. If additional ITP is applied, drug combinations were cisplatin (70–100 mg), adriamycin (30–50 mg) and mitomycin C (15–20 mg) per treatment cycle (5 to 12 min infusion time, treatment cycles in 3 weeks intervals). The specified dosages are total dosages.

#### Criteria for responses and adverse events

Tumor responses were assessed in accordance with Response Evaluation Criteria in Solid Tumors (RECIST version 1.1) at 2–8 weeks after every second treatment cycle. CT, Magnetic Resonance Imaging (MRI), and Positron Emission Tomography (PET) evaluated responses.

Pain controlled by < 50% analgesic administration 20 days after treatment was considered objective pain relief. Adverse events were assessed according to the common terminology criteria for adverse events of the national cancer institute.

### Statistical analysis

Statistics have been calculated with 95% confidence limits. Survival times were estimated using the Kaplan–Meier product limit estimator and follow-up for surviving patients was minimum 10 months, median follow-up was 39 months. Survival times were stratified according to clinical variables that may affect survival and logrank-tests were used to verify significance. Statistical analyses were performed using MediasStat software, version 28.5.14.

## Results

### Blood distribution in the tumor region of head and neck cancer patients

**Fig. 2:**
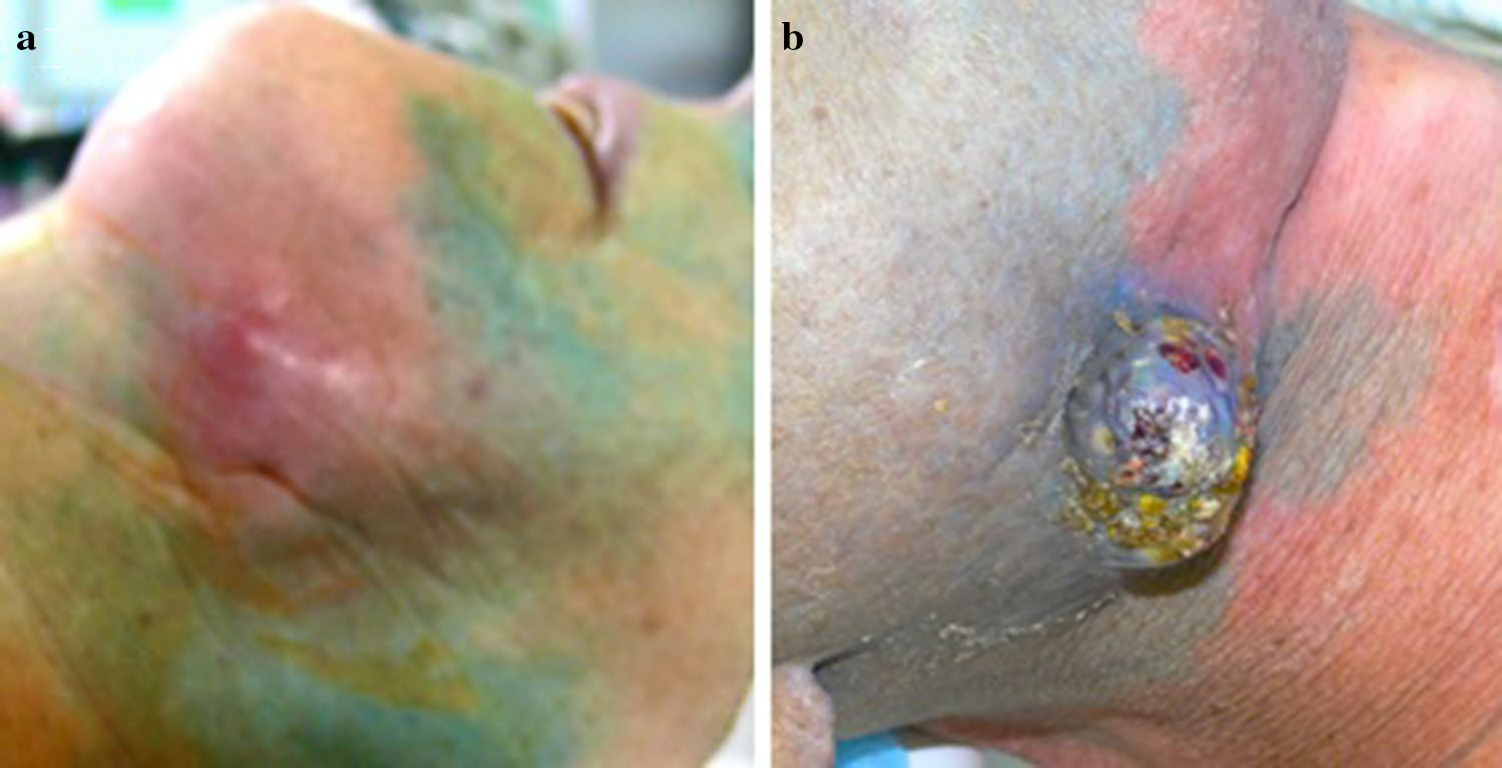
**a, b** Preirradiated areas do not stain after intra-arterial injection of blue dye, while non-irradiated and well-vascularized areas stain blue

The intra-arterial catheter was used for tissue staining by injecting indigocarmine blue. Regions with good blood supply showed distinct staining while regions with decreased blood supply showed little or no staining (Fig. 2a). Naturally, HNC exhibited high infiltrations of blood vessels, and therefore, are accessible for staining as well as chemotherapy (Fig. 2b).

Six–eight months after irradiation, connective tissue fibrosis affects the local blood supply, and the preirradiated area does not stain.

### Survival rates of HNC patients receiving short-term intra-arterial infusion chemotherapy are dependent on prior irradiation

**Fig. 3:**
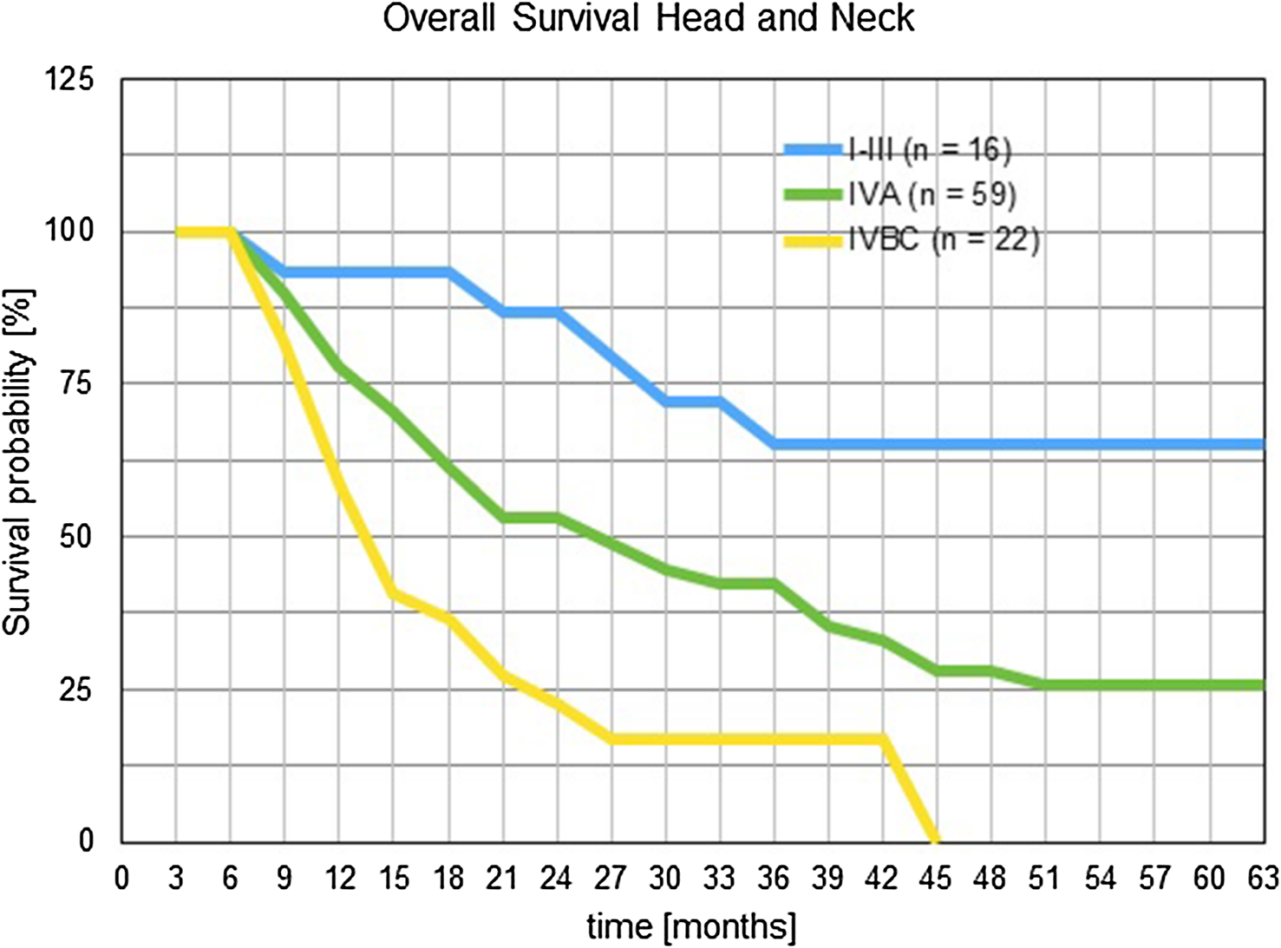
Survival times for head and neck cancer patients after short-term intra-arterial chemotherapy. Patients were clustered into staging groups and survival times were estimated with the Kaplan–Meier product limit estimator

Overall survival times have been estimated using the Kaplan–Meier products and staging groups have been clustered for reaching reasonable patient numbers. One-year survival was 59%, 82%, and 93%, respectively, for staging groups IV B/C, IVA and I–III. Two-year survival rates were 22%, 53% and 86% for the same staging groups. Three-year survival was 17%, 42%, and 65% for staging groups IV B/C, IVA and I–III (Fig. 3).

**Fig. 4:**
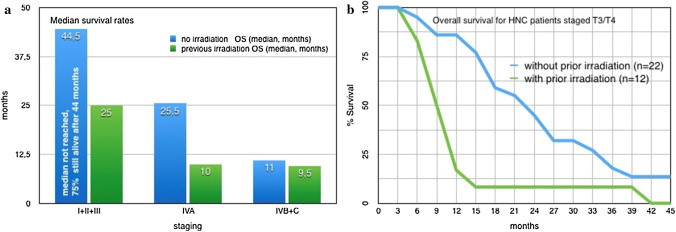
a and b: Median and overall survival rates for HNC patients with and without prior irradiation

The observed survival of HNC patients after intra-arterial infusion chemotherapy is strongly dependent on prior irradiation, and therefore, needs to be considered separately. After a median observation time of 39 months, 3 out of 35 patients with prior irradiation were still alive (since 11, 24, and 74 months), and 29 out of 62 patients without any pretreatment were still alive (median 39 months, range 10–221 months). The median survival for stage I–III patients without irradiation was not reached. After 44.5 months, 75% of the patients were still alive. The median survival of stages I–III and IVA was decreased for patients with prior irradiation. Survival times of stage I–III and stage IVA HNC groups, treated with short-term i.a. chemotherapy differed significantly if prior irradiation had been administered or not (*p* value < 0.01 and *p* value < 0.005).The median survival for stages IVB/C was slightly lower for patients with prior irradiation than for patients without pretreatment (9.5 vs 11 months) (Fig. 4a). Survival times showed a slightly positive effect for non-pretreated patients but without significance (*p* value < 0.6).

Importantly, if advanced cases with very large tumor masses of the primary tumor are considered, a divergence in the survival rates of patients with or without pretreatment can be detected. In patients who received prior radiation or no pretreatment, a tumor diameter of more than 4 cm (T3/T4) yields a median survival of 9 or 22.5 months, respectively (Fig. 4b).

### Response rates of HNC patients receiving short-term intra-arterial infusion chemotherapy

Response rates for HNC patients vary significantly with regard to prior radiation therapy. The response was evaluated in comparison to the results of the CT scan. The subgroup without any prior therapy (*n* = 62) responded better to short-term intra-arterial chemotherapy than the subgroup with prior radio- or radiochemotherapy (*n* = 35) in all stages. For the patients without pretreatment, 25%, 14%, and 0% cases of complete response were reached for stages I–III, IVA, and IVBC, respectively. In addition, 42%, 38%, and 68% of partial response were reached for the same staging groups. For the patients with prior radio- or radiochemotherapy, complete response was only reached for 6% of the stage IVA group and none of the other staging groups. Partial response was reached for 25%, 24%, and 29% for staging groups I–III, IVA, and IVBC, respectively (pretreated patients). A clinical picture of response is shown in the supplement figure.

### Adverse effects and quality of life

No cases of dysphagia, xerostomia, or neurological damage in terms of functional speech loss or ototoxicity were noted with the given drug combinations, dosages, and infusion times. No patient required a tracheostomy or tube feeding. The bone marrow depression was within the acceptable range of grade 2 for patients without pretreatment. The patients who had undergone prior chemoradiation with extensive doses of systemic chemotherapy exhibited WHO grade 3–4 bone marrow depression after intra-arterial exposure at moderate doses. Complete reductions in pain were yielded in 25% of stage IVB/C patients without pretreatment, and in 14% of stage IVB/C patients with prior irradiation.

## Discussion

Standard therapy of HNC consists of a combination of radiotherapy with high-dose intravenous chemotherapy but, despite good tumor control rates and long survival rates, outcomes remain unsatisfactory due to the severe side effects and poor quality of life. Three-year overall survival with standard therapy range between 25% and 75% depending on tumor size, extension, free surgical margins or relapse.

With intensity-modulated radiotherapy (IMRT), proton therapy and immune checkpoint inhibition, new treatment options have been established, especially in tumors that positively test for human papilloma virus. So far, no definitive data are available and the standard treatment with radiotherapy and high-dose chemotherapy is still used in most cases.

In specialized centers, however, short-term intra-arterial chemotherapy provided interesting results in phase II and III studies. Short-term intra-arterial infusion chemotherapy with chemofiltration has been shown to reach high cytostatic concentrations at the tumor site while maintaining low concentrations in the unaffected areas of the rest of the body. Adverse effects have shown to be correspondingly low. Good response rates can be reached even for very large tumor masses and in advanced staged patients. Intra-arterial infusion for HNC treatment has also been performed with different infusion times, drug dosages and combinations but never with additional isolated thoracic perfusion. This technique increases the local exposure to drugs (Ye et al. 2016; Suzuki et al. 2016).

Independent of genetic, molecular and virus infection data, short-term intra-arterial infusion chemotherapy is a noteworthy option for HNC patients. A good blood supply in the tumor is crucial for the response; if it is present, even very large tumor masses show a prompt response. Since blood supply is mostly reduced in preirradiated tumors, at approximately 6–8 months postirradiation, especially in tumors > 4 cm (T3/T4), a reduced response behavior even to i.a. chemotherapy is observed.

Limitations of this study are the missing data on human papillomavirus (HPV) status and the lack of homogeneity according to patient groups and treatment modalities. Preirradiation has been shown to affect the treatment response but radiation dosages are divergent. Detailed response rates according to treatment technique cannot be provided due to inhomogeneous patient characteristics and too small patient cohorts.

However, since these techniques outperform others and show high drug concentration rates at the tumor site while maintaining low drug concentrations in the healthy parts of the body, high response rates and low adverse effects, the techniques are worth considering not only for HPV-negative patients but also for other patients with head and neck cancer. A rearrangement of the possible treatment options for different patient groups should be made in terms of changing the sequence of treatments with priority given to the treatment that does not block possible further treatments and has a good chance of response.

Special attention should be given to the different kinds of intra-arterial application pathways. Infusion times of 5–12 min have empirically been shown to yield the highest tumor tissue concentrations, optimal drug exposure and best response rates (Aigner et al. 1988).

Drug exposure can be increased by means of intra-arterial infusion of slightly higher dosages with simultaneous chemofiltration of the venous return from the tumor site (Aigner et al. 1983, 2016). Maximally increased drug exposure is achieved when the intra-arterial infusion is combined with isolated perfusion techniques (Aigner et al. 2018; Guadagni et al. 2004, 2007).

The drug dosages for intra-arterial application in general can be lower than those required for intravenous application; nevertheless, i.a. application yields higher drug concentrations during the first pass through the tumor site. The intra-arterial bolus infusion with “systemic” dosages, however, by itself may generate toxic systemic drug levels. As shown in the Netherlands Cancer Institute’s randomized study of systemic versus chemoradiation for HNC, no study arm has a significant advantage with respect to survival. Even though it causes fewer side effects in the subject, intra-arterial angiographic chemotherapy requires much more effort than simple intravenous injection (Rasch et al. 2010).

Prior chemoradiotherapy has a negative influence on response to intra-arterial chemotherapy because of impaired blood supply due to connective tissue fibrosis. With regard to the good survival rates and consistent quality of life with nearly no toxicity, short-term intra-arterial chemotherapy for HNC could be considered as a first option in a treatment protocol, and if initial treatment fails, patients undergo irradiation (Aigner et al. 2018).

## Conclusions

Short-term intra-arterial infusion chemotherapy applied with an implanted port system or an angiocatheter and optionally combined with isolated thoracic perfusion seems to be an effective treatment option for HNC patients, even with very large tumor masses. This method results in good response rates while keeping adverse events low.

## Electronic supplementary material

Below is the link to the electronic supplementary material.

Supplement figure: Cancer of the tonsil before and four weeks after intra-arterial infusion chemotherapy (JPG 185 KB)

## References

[CR1] Achim V, Bolognone RK, Palmer AD et al. Long-term functional and quality-of-life outcomes after transoral robotic surgery in patients with oropharyngeal cancer. JAMA Otolaryngol Head Neck Surg 10.1001/jamaoto.2017.1790 (Online 2017) 10.1001/jamaoto.2017.1790 PMC5833591 29075740

[CR2] Aigner K, Tonn J, Hechtel R, Seuffer R (1983) Die Intraarterielle Zytostatikatherapie mit Venöser Filtration im Halboffenen System. Onkologie 6:74–76 6346193 10.1159/000215204

[CR3] Aigner K, Müller H, Walther H, Link K (1988) Drug filtration in high-dose regional chemotherapy. Regional cancer treatment. Contrib Oncol Basel Karger 29:261–280

[CR4] Aigner KR, Selak E, Schlaf R (2016) Isolated thoracic perfusion with carotid artery infusion for advanced and chemoresistant tumors of the parotid gland and tonsils. In: Aigner KR, Stephens FO (eds) Induction chemotherapy: systemic and locoregional. Springer International Publishing, Cham, pp 123–130

[CR5] Aigner KR, Gailhofer S, Aigner K (2018) Carotid artery infusion via implantable catheters for squamous cell carcinoma of the tonsils. World J Surg Oncol 16:104. 10.1186/s12957-018-1404-8 29871686 10.1186/s12957-018-1404-8 PMC5989445

[CR6] Anguiano L, Mayer DK, Piven ML, Rosenstein D (2012) a literature review of suicide in cancer patients. Cancer Nurs 35:E14–E26 21946906 10.1097/NCC.0b013e31822fc76c

[CR7] Briscoe J, Webb JA (2016) Scratching the surface of suicide in head and neck cancer. JAMA Otolaryngol Head Neck Surg 142:610–610 27031661 10.1001/jamaoto.2016.0255

[CR8] Green BH, Griffiths EC (2014) Hospital admission and community treatment of mental disorders in England from 1998 to 2012. Gen Hosp Psychiatry 36:442–448 24726764 10.1016/j.genhosppsych.2014.02.006

[CR9] Guadagni S, Müller H, Valenti M, Clementi M, Fiorentini G, Cantore M, Amicucci G (2004) Thoracic stop-flow perfusion in the treatment of refractory non-small cell lung cancer. J Chemother. 16(Suppl 5):40–43 15675476 10.1080/1120009x.2004.11782382

[CR10] Guadagni S, Clementi M, Valenti M, Fiorentini G, Cantore M, Kanavos E, Caterino GP, Di Giuro G, Amicucci G (2007) Hypoxic abdominal stop-flow perfusion in the treatment of advanced pancreatic cancer: a phase II evaluational trial. Eur J Surg Oncol 33:72–78 17166688 10.1016/j.ejso.2006.10.042

[CR11] Kovacs AF, Dobert N, Engels K (2012) The effect of intraarterial high-dose cisplatin on lymph nodes in oral and oropharyngeal cancer. Indian J Cancer 49:230–235 23107976 10.4103/0019-509X.102918

[CR12] Marta GN, Silva V, de Andrade Carvalho H et al (2014) Intensity-modulated radiation therapy for head and neck cancer: systematic review and meta-analysis. Radiother Oncol 110:9–15 24332675 10.1016/j.radonc.2013.11.010

[CR13] Misono S, Weiss NS, Fann JR, Redman M, Yueh B (2008) Incidence of suicide in persons with cancer. J Clin Oncol 26:4731–4738 18695257 10.1200/JCO.2007.13.8941 PMC2653137

[CR14] Osazuwa-Peters N, Boakye EA, Walker RJ, Varvares MA (2016) Suicide: a major threat to head and neck cancer survivorship. J Clin Oncol 34:1151 26786926 10.1200/JCO.2015.65.4673

[CR15] Park RC, Kam D, Salib A et al (2016) Scratching the surface of suicide in head and neck cancer-reply. JAMA Otolaryngol Head Neck Surg 142:611 27031774 10.1001/jamaoto.2016.0258

[CR16] Rasch CR, Hauptmann M, Schornagel J et al (2010) Intra-arterial versus intravenous chemoradiation for advanced head and neck cancer: results of a randomized phase 3 trial. Cancer 116:2159–2165 20187094 10.1002/cncr.24916

[CR17] Rathod S, Livergant J, Klein J, Witterick I, Ringash J (2015) A systematic review of quality of life in head and neck cancer treated with surgery with or without adjuvant treatment. Oral Oncol 51:888–900 26209066 10.1016/j.oraloncology.2015.07.002

[CR18] Ringash J (2015) Survivorship and quality of life in head and neck cancer. J Clin Oncol 33:3322–3327 26351336 10.1200/JCO.2015.61.4115

[CR19] Ringash J, Bernstein LJ, Cella D et al (2015) Outcomes toolbox for head and neck cancer research. Head Neck 37:425–439 24318166 10.1002/hed.23561

[CR20] Robbins K, Howell S, Williams J (2010) Intra-arterial chemotherapy for head and neck cancer: is there a verdict? Cancer 116:2068–2070 20186833 10.1002/cncr.24930

[CR21] Suzuki T, Sakashita T, Homma A, Hatakeyama H, Kano S, Mizumachi T, Yoshida D, Fujima N, Onimaru R, Tsuchiya K, Yasuda K, Shirato H, Suzuki F, Fukuda S (2016 Oct) Effectiveness of superselective intra-arterial chemoradiotherapy targeting retropharyngeal lymph node metastasis. Eur Arch Otorhinolaryngol 273(10):3331–3336. 10.1007/s00405-016-3933-5**(Epub 2016 Feb 13)** 26874732 10.1007/s00405-016-3933-5

[CR22] Ye M, Li X, Liu W, Tao H, Yan JJ (2016) Evaluating the efficacy and safety of continuous arterial infusion chemotherapy with cisplatin and 5-fluorouracil in treating oral cancer. Cancer Res Ther 12(Supplement):47–49. 10.4103/0973-1482.191629 10.4103/0973-1482.191629 27721252

[CR23] Zecha JA, Raber-Durlacher JE, Nair RG et al (2016) Low level laser therapy photobiomodulation in the management of side effects of chemoradiation therapy in head and neck cancer: part 1: mechanisms of action, dosimetric, and safety considerations. Support Care Cancer 24:2781–2792 26984240 10.1007/s00520-016-3152-z PMC4846477

