# Supplementary figures and images for: Short-term intra-arterial infusion chemotherapy for head and neck cancer patients maintaining quality of life
Source: J Cancer Res Clin Oncol. 2018 Oct 31;145(1):261–8. doi: 10.1007/s00432-018-2784-4 (PMC6325995; doi:10.1007/s00432-018-2784-4)

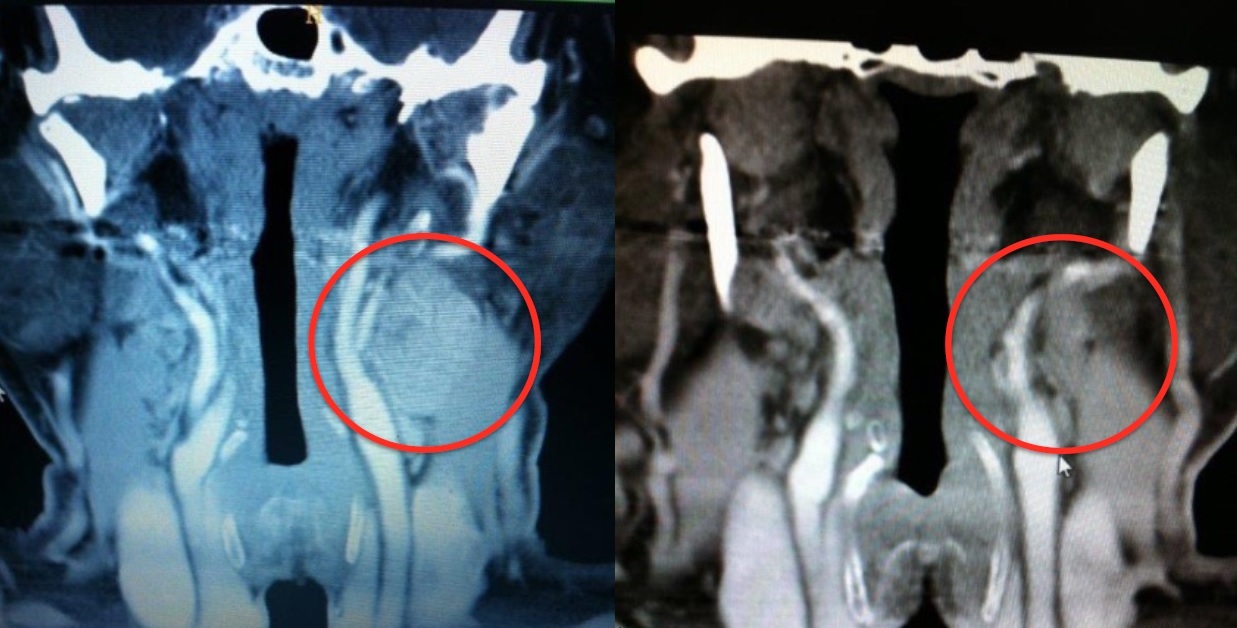

Supplement: Supplementary file 1 — Supplement figure: Cancer of the tonsil before and four weeks after intra-arterial infusion chemotherapy (JPG 185 KB) [file 432_2018_2784_MOESM1_ESM.jpg]
